# EEG slow-wave coherence changes in propofol-induced general anesthesia: experiment and theory

**DOI:** 10.3389/fnsys.2014.00215

**Published:** 2014-10-29

**Authors:** Kaier Wang, Moira L. Steyn-Ross, D. A. Steyn-Ross, Marcus T. Wilson, Jamie W. Sleigh

**Affiliations:** ^1^School of Engineering, The University of WaikatoHamilton, New Zealand; ^2^Waikato Clinical School, The University of Auckland, Waikato HospitalHamilton, New Zealand

**Keywords:** slow-wave sleep, phase-coherence measure, mean-field cortical model, gap-junction, Turing–Hopf instabilities

## Abstract

The electroencephalogram (EEG) patterns recorded during general anesthetic-induced coma are closely similar to those seen during slow-wave sleep, the deepest stage of natural sleep; both states show patterns dominated by large amplitude slow waves. Slow oscillations are believed to be important for memory consolidation during natural sleep. Tracking the emergence of slow-wave oscillations during transition to unconsciousness may help us to identify drug-induced alterations of the underlying brain state, and provide insight into the mechanisms of general anesthesia. Although cellular-based mechanisms have been proposed, the origin of the slow oscillation has not yet been unambiguously established. A recent theoretical study by Steyn-Ross et al. (2013) proposes that the slow oscillation is a network, rather than cellular phenomenon. Modeling anesthesia as a moderate reduction in gap-junction interneuronal coupling, they predict an unconscious state signposted by emergent low-frequency oscillations with chaotic dynamics in space and time. They suggest that anesthetic slow-waves arise from a competitive interaction between symmetry-breaking instabilities in space (Turing) and time (Hopf), modulated by gap-junction coupling strength. A significant prediction of their model is that EEG phase coherence will decrease as the cortex transits from Turing–Hopf balance (wake) to Hopf-dominated chaotic slow-waves (unconsciousness). Here, we investigate changes in phase coherence during induction of general anesthesia. After examining 128-channel EEG traces recorded from five volunteers undergoing propofol anesthesia, we report a significant drop in sub-delta band (0.05–1.5 Hz) slow-wave coherence between frontal, occipital, and frontal–occipital electrode pairs, with the most pronounced wake-vs.-unconscious coherence changes occurring at the frontal cortex.

## 1. Introduction

General anesthetic drugs act to suppress the conscious state of the cortex, leading it to a natural sleep-like mode (Lancel, 1999; Franks, 2008). There is clinical evidence showing that such sedated unconsciousness can be induced by the injection of anesthetic substances into some discrete brain areas which are critical in the coordination of sleep-wake transitions (Sukhotinsky et al., 2007). Further evidence to support the notion of strong similarity between natural deep sleep and anesthesia can be seen in the electrical activity of the cortex: both states are signposted by the abrupt onset of large, slow oscillations (0.1–1.5 Hz) in the electroencephalogram (EEG) and local field potential (Steriade et al., 1993). These rhythmic signals, which sweep through the brain during deep sleep at the rate of about 1 cycle per second (Massimini et al., 2004), have been shown to play a role in memory encoding and consolidation (Steriade and Timofeev, 2002; Walker, 2009).

Although EEG slow waves are manifest in an unconscious state, they are also superimposed on the alpha and theta waves when our brain is in a low conscious level, the so-called “idling” state where the brain is not engaged in the active processing of information (Uusberg et al., 2013). Clinical studies show a stable increase in power of the lowest frequency components of the EEG signal as anesthesia deepens, while higher frequency components (theta, alpha, gamma) are highly variable during and after loss of consciousness (Sleigh et al., 2000; Lewis et al., 2012). Thus, tracking the emergence of slow-wave oscillations during transition to unconsciousness may help us to identify drug-induced alterations of the underlying brain state, and provide insight into the mechanisms of general anesthesia.

In the last decades, there has been a growing understanding of how slow waves are generated during sleep. Steriade et al. (1989) reported slow-wave activity (SWA) from *in vitro* thalamic slices. In thalamocortical (TC) neurons, SWA depends on voltage-sensitive properties of low-threshold calcium channels [known as “T” type (David et al., 2013)] that may provide a pacemaking role, mediating the transition between tonic firing and low-threshold spiking (Suzuki and Rogawski, 1989; Astori et al., 2011). However, the “clock-like” SWA generated by TC neurons is more regular than that of slow-wave sleep (Nir et al., 2010). Further, it is known that *in vitro* cortical slices can produce slow oscillations of local field potential in the absence of thalamic inputs (McCormick and Sanchez-Vives, 2000). So slow rhythmic thalamic activity may not be relevant to the onset of slow cortical waves.

Human EEG recordings show that the slow oscillations seem to originate from nearly any region of the scalp and behave as a traveling wave propagating in any direction (Massimini et al., 2004). Yet, recent clinical studies demonstrate that the slow waves can be locally regulated (Huber et al., 2004, 2006; Murphy et al., 2009). Therefore, questions remain about where slow waves originate and whether all cortical areas engage equally in slow-wave activity.

To help address this deficit, Steyn-Ross et al. (2013) presented a physiologically-motivated mathematical model of the cortex that demonstrates how coupling via inhibitory electrical synapses (gap-junctions) mediates the generation of propofol anesthetic slow waves. The model envisions the cortex as a *mean-field* continuum in which pools of neurons are linked via chemical and electrical synapses. GABAergic anesthetic agents, such as propofol, act at chemical synapses to hyperpolarize postsynaptic neurons by prolonging the duration of the inhibitory postsynaptic potential (IPSP) via increased influx of chloride ions (Franks and Lieb, 1994; Kitamura et al., 2003). In addition to chemical neuromodulation, there is evidence that propofol reduces the resistive gap-junction coupling between adjoining inhibitory neurons (Wentlandt et al., 2006; Huang et al., 2014) that is proposed to form a broad diffusive syncytium linking inhibitory neural populations (Fukuda et al., 2006). Accordingly, we model anesthetic effect as a moderate reduction in inhibitory diffusion, paired with an increase in inhibitory postsynaptic potential. In the vicinity of a general-anesthetic induced transition from wake to coma, the Steyn-Ross model describes a subtle rebalancing of cortical Turing (spatial) and Hopf (temporal) instabilities to an unconscious state that is characterized by Hopf-dominated slow waves whose dynamics is chaotic in time and space.

Identifying the specific dynamics of slow waves associated with loss of consciousness requires an examination of the transition into unconsciousness. In this paper, we examine the clinical EEG recordings in terms of slow-wave phase-coherence between different electrode-pairs, comparing coherence values before and after the induction of propofol anesthetic. Propofol, a widely used anesthetic drug, enhances GABAergic inhibitory input to neurons (Bai et al., 1999; Rudolph and Antkowiak, 2004), with effects in cortex, brainstem, thalamus and spinal cord (Fiset et al., 1999; Kungys et al., 2009). EEG coherence is considered to be a qualitative measure of the degree of association or coupling between two EEG channels. Coherence estimation for high-density EEG recording is able to demonstrate functional cooperation between two brain regions (Nunez and Srinivasan, 2006), revealing subtle changes in brain dynamics. We compare our findings with a testable prediction by Steyn-Ross et al. (2013) and illustrated here in **Figures 9E, 10E, 11** (compare “non-cognitive wake” with “anesthetic slow-wave”): namely, introduction of anesthetic to the awake brain should lead to a significant *decline* in low-frequency EEG phase-synchrony.

## 2. Materials

The EEG dataset used in this study are archived files from Waikato Clinical School, Hamilton, New Zealand, previously used to investigate anesthetic response of EEG across different frequency bands (Johnson et al., 2003). The dataset contains pairs of 60-s EEG (sampling frequency 250 Hz) recordings for two distinct well-developed brain states: wake and propofol anesthetic coma, recorded from 5 healthy adult subjects via 129 electrodes^1^ using an EGI™ dense array with Cz (vertex) being the reference electrode. The archival EEG dataset are manually selected epochs that are relatively artifact-free.

**Figure 1:**
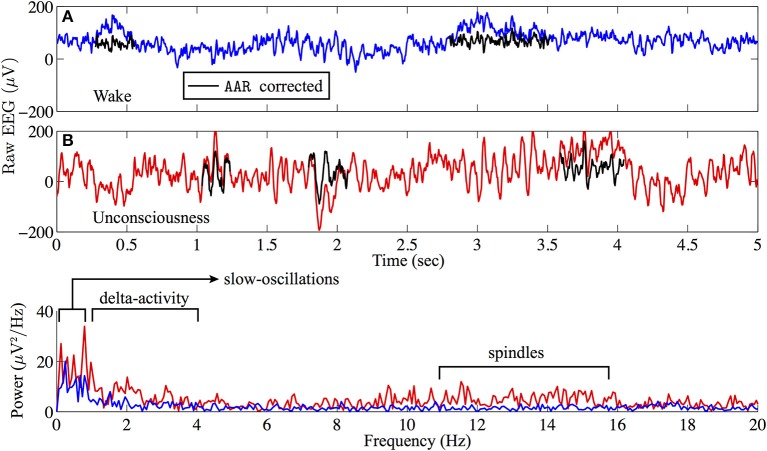
**Sample (A) wake (blue) and (B) sedated unconsciousness (red) EEG from archival Fp1 recording**. Raw EEG data are filtered via AAR^2^ to remove eye-blink artifacts. AAR-corrected EEG are marked in black. The power spectra show that slow-wave oscillations are dominant in the sedated unconsciousness state.

**Figure 2:**
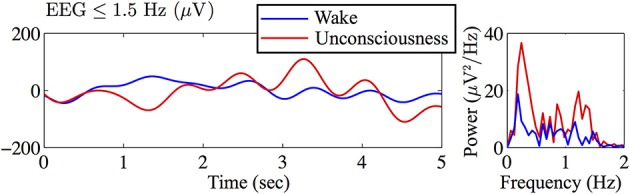
**Filtered Figure 1 EEG in sub-delta band (≤1.5 Hz) and corresponding power spectra (computed by Matlabfft) revealing a strong slow-wave (~0.3 Hz) in the sedated unconsciousness state**.

An example of EEG recorded from electrode Fp1 is represented in Figure 1. This demonstrates the clear contrast between wakefulness (upper EEG trace) and sedated unconsciousness (lower trace) with the appearance of spindles (12–15 Hz) and slow rhythms including delta activity (1–4 Hz) and slow oscillations (0.2–1 Hz). By focusing on the EEG in sub-delta band (≤1.5 Hz), Figure 2 shows that the power of the slow-waves in sedated unconsciousness is nearly twice as large as that in the wake state.

## 3. Methods

### 3.1. Measuring EEG coherence

EEG coherence between two electrode sites is usually computed by one of two methods: the Fourier transform (FT) cross spectrum (Achermann and Borbely, 1998), or the Hilbert transform (HT) instantaneous phase difference (Mormann et al., 2000) between two EEG time-series.

Since EEG represents the activities of the non-linearly interacting neuronal populations, it is neither truly linear nor stationary. Thus, it may be unreliable to use FT-based methods for EEG analysis since these assume that the time-series is stationary (Lo et al., 2009; Zhang et al., 2010).

The Hilbert transform (Huang et al., 1998; Sweeney-Reed and Nasuto, 2007) circumvents the requirement for stationarity by generating an analytic signal to extract the instantaneous frequency and phase angle from the original non-stationary signal. The mean of the phase divergence between two time-series yields an index characterizing the phase synchronization between them. The advantages of the HT over the traditional FT-based approaches have been appreciated in many studies of cortical neuronal synchronization under different circumstances such as Parkinson's disease (Tass et al., 1998), abrupt seizure (Oweis and Abdulhay, 2011), sleep (Yi et al., 2009), and anesthetic coma (Koskinen et al., 2001).

### 3.2. Hilbert transform

A real time-series *X*(*t*) can be transformed to a complex function known as the analytic signal:

(1) $$\hat{X}(t)=X_{r}(t)+iX_{i}(t)$$

where *X*_*r*_(*t*) is the original series *X*(*t*) and *X*_*i*_(*t*) is the Hilbert transform of *X*(*t*) (Mormann et al., 2000; Koskinen et al., 2001). The instantaneous phase of *X*(*t*) is computed by:

(2) $$\phi (t)={\text{tan}}^{-1}\left(\frac{X_{i}(t)}{X_{r}(t)}\right)$$

To quantify the phase synchronization between two time-series *X*_*m*_(*t*) and *X*_*n*_(*t*), a coherence index based on work by Kuramoto (Kuramoto, 1984; Kuramoto and Nishikawa, 1987) is used:

(3) $$R_{\left(m,n\right)}\;=\;|\langle e^{i[\phi_{m}(t)-\phi_{n}(t)]}\rangle |$$

The mean phase coherence *R* measures the time-averaged phasor for the angular distribution of the phase difference between the two time-series; *R* lies between 0 and 1, with 1 representing perfect phase coupling. This style of Kuramoto order-parameter has been widely used in the study of synchronization dynamics (e.g., Acebrón et al., 2005; Steyn-Ross et al., 2013).

**Figure 3:**
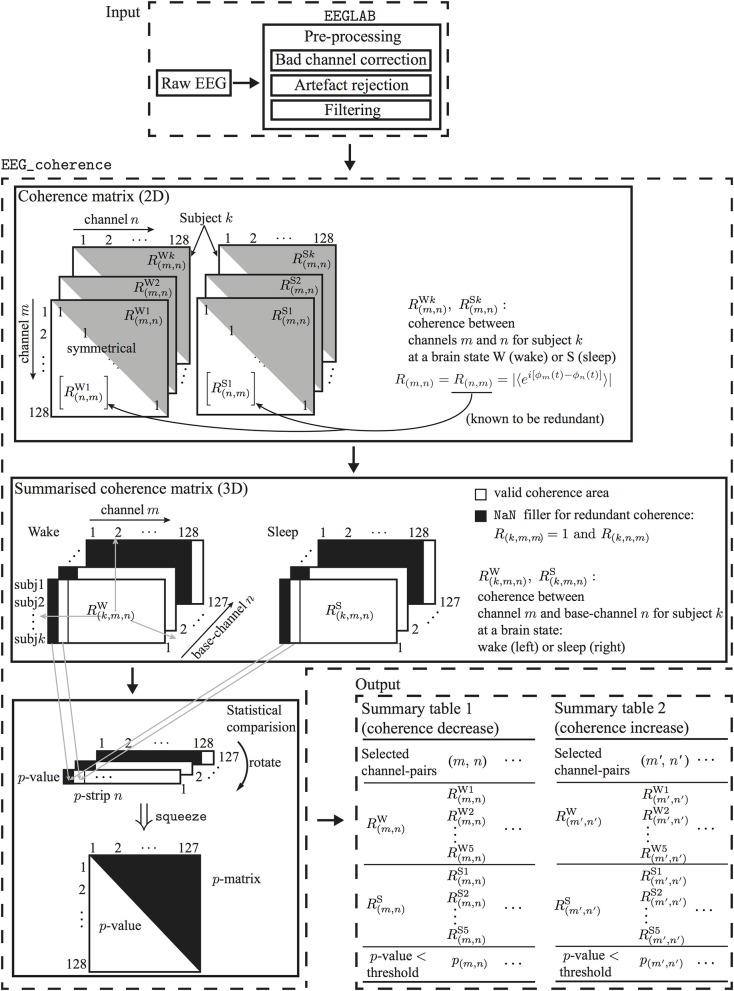
**Flowchart for processing EEG of two brain states to determine electrode-pairs with significantly altered phase-coherence**. EEG data undergo preprocessing in EEGLAB before passing to EEG_coherence, a customized Matlab algorithm that automatically identifies electrode-pairs with significantly altered phase-coherence between two brain states across multiple subjects, then stores these electrode-pair results in a summary table.

A Matlab implementation for computing the mean phase coherence between two signals reads as follows Steyn-Ross et al. (2012):


% Compute analytic (complex) signals for Xm and Xn
  Xmc = hilbert(Xm); Xnc = hilbert(Xn);
% Extract instantaneous phase angles
  phi_Xm = angle(Xmc); phi_Xn = angle(Xnc);
% Measure the average phase-coherence
  R = abs(mean(exp( 1i*(phi_Xm - phi_Xn))));

Let *X*_*m*_(*t*) and *X*_*n*_(*t*) be a pair of EEG recordings, respectively, from the electrodes *m* and *n*. A 129-channel EEG recording has, in principle, a total of 128 × 128 pairs of *R*-values (excluding the reference channel), but half of these are redundant since *R*_(*m*,*n*)_ = *R*_(*n*,*m*)_. The coherence matrix is represented as an *m* × *n* = 128 × 128 square grid with the unit diagonal [*R*_(*m*,*n*)_ = 1 when *m* = *n*], which separates the matrix into two symmetrical triangles [*R*_(*m*,*n*)_ = *R*_(*n*,*m*)_]. Practically, we need only examine the upper triangle [i.e., *R*_(*m*,*n*)_] of the *R* matrix. See Figures 3, **9E** for an illustration of the structure of the coherence matrix.

For coherence calculations, we use a 5-s moving window with 1-s overlap, and follow Mormann et al. (2000) and Steyn-Ross et al. (2012, 2013) in applying a Hann window, retaining only the middle 80% of each segment to minimize edge distortions from the Hilbert transform. The final determined coherence is the average of those obtained from the windowed signal segments. We repeated the coherence calculations using longer windows, including the full 60-s extent, and found no significant changes, so we concluded that, provided the brain dynamical state does not vary dramatically during the windowed interval, the sub-delta coherence measure is not particularly sensitive to window size.

### 3.3. EEG_coherence: an automatic EEG processing algorithm for EEG coherence analysis

The raw EEG data were visually inspected and the artifacts were manually marked using EEGLAB^3^ (Delorme and Makeig, 2004). The one or two bad channels were replaced by substituting with the average of the four neighboring channels. Eye-blink artifacts were removed using AAR (see Figure 1 for definition and details), then the repaired traces were inspected for smoothness and continuity. Since the archival EEG data are relatively artifact-free, only minor corrections were needed. We filtered EEG to the sub-delta band (≤1.5 Hz) using EEGLAB built-in basic FIR (linear finite impulse response) order-2 filter with the pass-band between 0.05 and 1.5 Hz. During filtering, EEGLAB uses the Matlab routine filtfilt() to apply the filter forward and then backward, ensuring that phase delays introduced by the filter are nullified. The resulting sub-delta band EEG traces show characteristic slow oscillations; this is the prominent feature of EEG activity during non-rapid eye movement (non-REM) sleep in humans (see Figure 2 for an example) (Marshall et al., 2003).

The EEGLAB pre-processed EEG data were then passed to EEG_coherence, a custom Matlab algorithm that identifies electrode-pairs with significantly altered phase-coherence between the two brain states. The user specifies the folder location where the EEG data are stored and configures some basic parameters (e.g., window and overlap length for the coherence measure). EEG_coherence automatically generates a summary table including identified electrode-pairs and their corresponding phase-coherence indices at two distinct brain states for all subjects. *p*-values that are used to identify those electrode-pairs whose phase-coherence has significantly altered are included in the table to permit further statistical analysis.

As shown in Figure 3, EEG_coherence processes EEG data in three steps:

1. Construction of coherence matrices: The phase-coherence measure is based on the Hilbert transform, as described in Section 3.2. Each subject will have two coherence matrices, awake and sleep, for the wake and unconscious states, respectively.
2. Extraction of coherence summaries: For each brain state (wake or unconsciousness), EEG_coherence will construct a consolidated tableau of matrices by concatenating the coherence matrices for all five subjects. This consolidated table has three dimensions: the first dimension (row-index *k* = 1 … 5) points to the subject, while the second (channel-index *m* = 1 … 128), and third dimensions (base-channel index *n* = 1 … 127) identify the specific pair of electrodes whose phase similarity is being assessed. Thus, coordinate (*k*, *m*, *n*) captures the coherence *R*_(*k*,*m*,*n*)_ between EEG channels *m* and *n* for subject *k*. Since we only consider the upper triangle of the coherence matrix, the redundant coherence entries^4^ in the summary matrix will be filled with NaN (not a number). The output from this step is a pair of coherence summary matrices for wake and unconscious states.
3. Statistical comparison: A one-tail Mann-Whitney *U*-test is performed to test the null hypothesis *H*_0_ that the five pairs of wake/sleep coherence values—at a given (*m*, *n*) matrix coordinate—are drawn from populations with *equal* medians against the alternative that they are not. With reference to Figure 3, this means that we are comparing the median of the 5 ×1 column-vector for wake [*R*^W^_(1,*m*,*n*)_, *R*^W^_(2,*m*,*n*)_, · · ·, *R*^W^_(5,*m*,*n*)_]^T^ against the median for the corresponding vector for sleep [*R*^S^_(1,*m*,*n*)_, *R*^S^_(2,*m*,*n*)_, · · ·, *R*^S^_(5,*m*,*n*)_]^T^. This comparison is repeated across all non-redundant channel pairs.
  In fact, the Mann-Whitney calculation is run twice to allow for testing against two distinct alternative hypotheses; namely, *H*_1_: that the median coherence is *higher* in wake than in sleep (i.e., right-tailed test), and, *H*_2_: that the median coherence is *lower* in wake than in sleep (left-tailed).
  The statistical comparison for a base-channel *n* returns a three-dimensional matrix named *p*-strip; this matrix contains *p*-values for channel-pairs 1-*n*, 2-*n*,…,128-*n*. The *p*-strip matrices are generated via the following Matlab implementation:
  
awake_size = size(awake);
prop_size = size(sleep);

% Check if two coherence matrices have the same
  size
if ~isempty(find((awake == sleep)==0))
    error(‘unequal size’);
end

% Create p-strip matrix
for base_ch = 1: size(awake, 3)
    for ch_ind = 1: size(awake, 2)
        if isnan(awake(:,ch_ind, base_ch))
            p(:,ch_ind, base_ch) = NaN;
        else
            [p(:,ch_ind, base_ch), h(:,ch_ind,
              base_ch)]…
            = ranksum(awake(:,ch_ind,
              base_ch),
                 sleep(:,ch_ind, base_ch),…
                 ‘alpha’, p_limit, ‘tail’,
                   direction);
            % direction: left: wake < sleep;
              right: wake > sleep
        end
    end
end
% Squeeze the 3D p-strip matrix, leading to a 2D
  p-matrix
p_matrix = squeeze(p);
    % e.g. E1-E2 is at row 2 (channel), col 1
     (base-channel)
  If, across all subjects, a given electrode-pair shows a statistically significant difference in coherence between wake and unconscious state (i.e., *p* < p_limit), EEG_coherence will store this electrode-pair in the summary table.

## 4. Results

### 4.1. Sub-delta EEG coherence changes across five subjects

We first examine the across-subject wake-vs.-sleep changes in sub-delta phase coherence using the methodology described in the previous section; then in Section 4.2 we analyze the coherence matrices for each individual subject.

**Figure 4:**
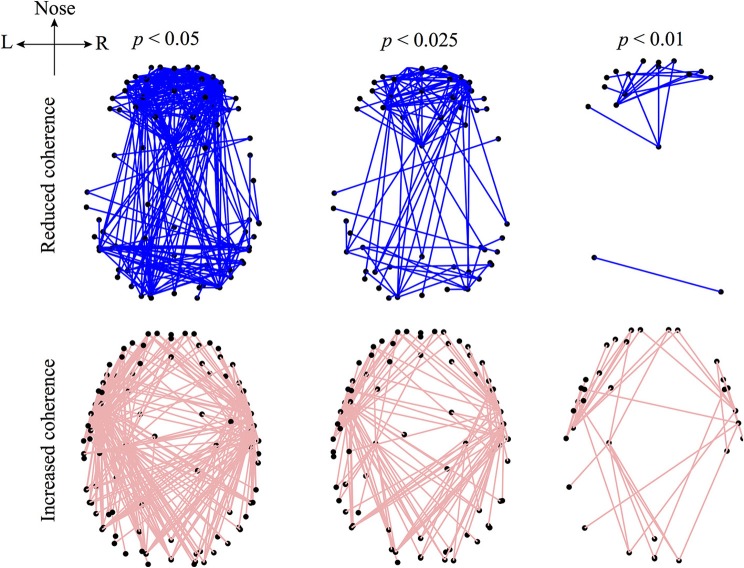
**Graphical representations of the electrode pairs with significantly *reduced* (upper panel) or *increased* (lower panel) phase-coherence of the sub-delta band (0.05–1.5 Hz) EEG induced by propofol anesthesia**. EEG data (128-channel recording) were recorded from 5 subjects and processed by the EEG_coherence algorithm diagrammed in Figure 3. The electrode pairs with significant (*p* < 0.05) changes in phase coherence are connected with lines. The electrode-pair map is represented in a bird's-eye view of the 3D head model (created via the modified EEGLAB function plotchans3d). The black dots are EEG_coherence selected electrodes. Electrode pairs for altered phase coherence are determined with different levels of significance (significance-level *p* was set at 0.05, 0.025, and 0.01 in the Mann-Whitney *U*-test). Smaller *p* thresholds result in a lower density of electrode-pair cluster due to the stricter selection criterion, however, the electrode-pair distributions are generally preserved in trend.

Figure 4 visualizes those electrode-pairs identified by EEG_coherence as having significantly altered (i.e., decreased or increased) coherence between wake and unconscious states. The comparison between the upper and lower panels of Figure 4 reveals two major features of the coherence changes with respect to propofol anesthesia:

- Decreased coherence for frontal, occipital, and frontal–occipital electrode-pairs: The electrode-pairs showing significantly reduced coherence form dense clusters for pairs lying within the frontal area of the cortex, within the occipital area, and also for pairs spanning the frontal–occipital scalp sites. These observations suggest that neuronal activities within frontal cortex and within occipital cortex, and cooperative behavior between them, are less strongly coupled when the brain is switched to the unconscious state. Scanning the top panels of Figure 4 from left to right, we see that the front electrodes manifest the most robust decreases in phase coherence, indicating that propofol anesthesia leads to increased disorder in neuronal activity in the frontal cortex.
- Increased coherence for left- and right-temporal electrode-pairs: Electrodes at the left- and right-temporal areas detect enhanced coherence. These maps of enhanced connectivities seem to be complementary to the preceding maps showing decreased frontal–occipital connectivity: coherence trends have been reversed with the significant front–back *uncoupling* (top panel) occurring simultaneously with a left–right *coupling*. Examining the lower panels of Figure 4, we see evidence of strengthened left–right electrode connectivity, showing increased EEG coherence with the induction of propofol anesthesia.

If we overlap the upper and lower panels of Figure 4, we find some frontal electrodes have decreased coherence with the occipital electrodes, while having increased coherence with the left- and right-temporal electrodes. Similarly, some occipital electrodes have decreased coherence with the electrodes in the frontal area, while having increased coherence with those in the temporal areas. These observations suggest an underlying compensatory mechanism between a subsystem of fronto–occipital and other cortical regions at sub-delta frequencies. Cantero et al. (2002) reported a similar compensatory phenomenon in coherence between the temporal and other cortical regions for the alpha (8–12 Hz) and sleep spindle (12–15 Hz) frequency ranges.

**Figure 5:**
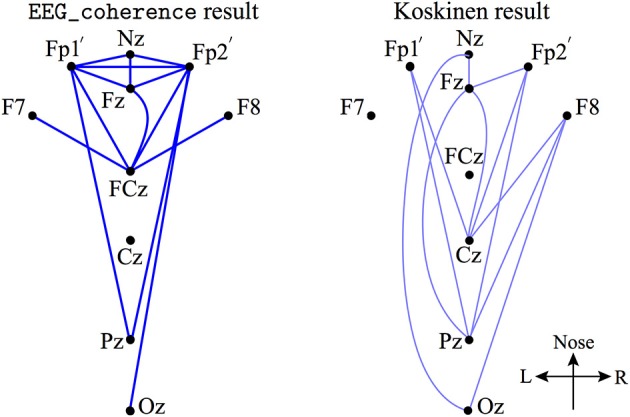
**A subset of electrode-pairs (left) showing significant (*p* < 0.05) reduction in phase coherence extracted from the upper left corner plot of Figure 4 (referenced to Cz, in dark blue lines) and Koskinen et al. reported pattern (Koskinen et al., 2001) (right, referenced to FCz, in light blue lines) for the coherence measured from 9 electrodes: Nz (nasion), Fp1′(about 1 cm down from Fp1, just above the eyebrow), Fp2′, Fz, F7, F8, Cz, Pz, and Oz**.

Furthermore, we examined the decreased EEG coherence patterns across nine electrodes (see the description of Figure 5) that Koskinen et al. utilized in their work (Koskinen et al., 2001), in which systematic phase synchronization changes were evaluated between EEG channel-pairs in various frequency bands during induction and recovery from propofol anesthesia. Koskinen et al. detected passband-specific behaviors in these changes, and identified a sub-delta EEG coherence decrease due to propofol-induced anesthesia. We set the significance level (*p* < 0.05) in EEG_coherence to be the same as that used by Koskinen et al. The comparison shown in Figure 5 illustrates that EEG_coherence produced a similar electrode-pair distribution pattern to the Koskinen findings, reinforcing our observation of sub-delta EEG coherence reduction in the frontal cortex. However, we need to add the caveat that the choice of reference electrode (Cz for the Koskinen recordings; FCz for the Waikato data) is reversed between the two experiments; fortunately these sites are adjacent on the scalp centerline, so can be expected to result in closely similar EEG traces.

We must acknowledge the possibility that the coherence changes we have detected may simply be the result of randomness: of the ~8000 network connections, by chance we can expect about 400 to show significant change at the uncorrected *p* = 0.05 level (1 in 20). To reduce the possibility of spurious significance (false positives), one could apply some form of *p*-value correction (such as Bonferroni) to compensate for multiple testing, but it is not clear how to do this straightforwardly with only five subjects. This motivates us to apply a clustering analysis to the individual coherence-change patterns as an alternative way of demonstrating robustness of our results.

### 4.2. EEG coherence changes for individual subjects

The coherence changes described in the previous section represent a population response across multiple subjects. Here, we present a much simpler analysis of the coherence changes for each of the five individuals, and show that the resulting clustering patterns are highly unlikely to have arisen by chance.

**Figure 6:**
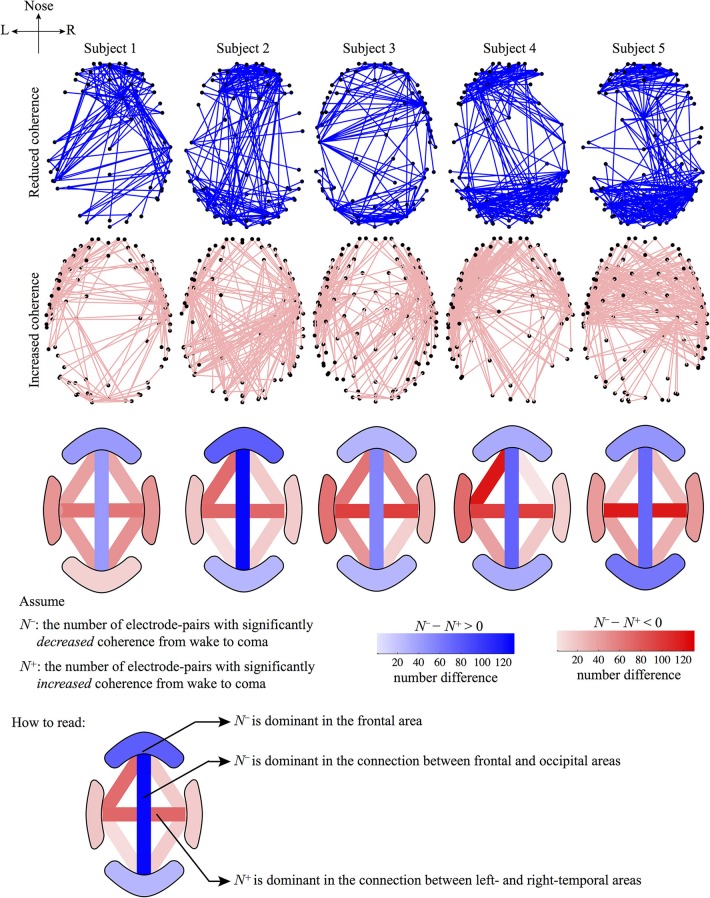
**Graphical representations of the electrode-pairs with significantly altered coherence from wake to coma for five subjects**. The first and second rows represent electrode-pairs with significantly *reduced* (blue lines) or *increased* (pink lines) coherence, respectively: selected electrode-pairs correspond to the top 5% most changed (i.e., most increased or most decreased) coherence during the wake to coma transition. The third row describes the number difference of electrode-pairs between the first and second rows for four regions: frontal, occipital, left- and right-temporal; and for six pair-wise connections *between* regions: frontal–left temporal, frontal–right temporal, frontal–occipital, left temporal–occipital, right temporal–occipital, left–right temporal. The number of electrode-pairs with significantly reduced (or increased) coherence in a region is counted as *N*^−^ (*N*^+^). The sign of (*N*^−^ − *N*^+^) determines the dominance of a coherence trend: if (*N*^−^ − *N*^+^) > 0, the region will be colored blue (decreased coherence); otherwise if (*N*^−^ − *N*^+^) < 0, the region will be colored red (increased coherence). The (*N*^−^ − *N*^+^) difference is calibrated by the color-gradient bar. (Note that the color-bar for the third row is not related to the first and second rows.)

The top two rows of Figure 6 are generated by a simple ranking of the (wake minus sleep) coherence differences for each individual. The first row shows the 5% of electrode-pairs exhibiting the largest positive difference (i.e., coherence *decreased* in sleep); the second row shows the 5% of electrode-pairs with the largest negative difference (i.e., coherence *increased* in sleep). We see that the spatial distribution of electrode-pairs with significantly altered coherence is generally preserved across the five subjects. The first row reveals clusters of electrode-pairs in the frontal and occipital areas with significantly *decreased* coherence; the second row shows the dense pairing of left–right electrodes with *increased* coherence along the temporal axis.

To quantify the coherence changes in specific areas of the cortex, we counted the number of electrode-pairs in the frontal region showing significantly *decreased* coherence (*N*^−^) and subtracted this from the number of frontal pairs with significantly *increased* coherence (*N*^+^). The difference (*N*^−^ − *N*^+^) is strongly positive (third row of figure), confirming that *N*^−^ (coherence decrease) is dominant in the frontal area. An opposite conclusion is reached for the left–right temporal electrode-pairs: (*N*^−^ − *N*^+^) is strongly negative with *N*^+^ being dominant (coherence increase), implying strengthened regional connections between hemispheres under anesthesia. We repeated these number difference calculation for ten cortical regions (see row 3). Blue (pink) shading indicates *N*^−^ (*N*^+^) dominance in a given cortical region.

**Figure 7:**
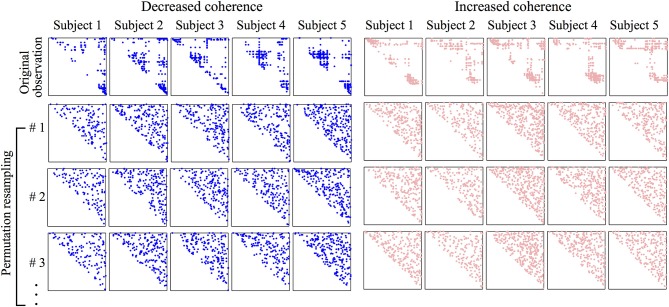
**Coherence matrices showing spatial distribution of electrode-pairs with significant wake vs. coma coherence difference**. The coherence matrix is diagonally symmetric, we need only display its upper half. The first row of the left panel corresponds to the first row of Figure 6; the first row of the right panel corresponds to the second row of Figure 6. The marked (either in blue or pink) dots in the matrix are the top 5% most changed (decreased: blue; increased: pink) phase-coherence during transit from wake to coma. The row and column indices of a marked dot identify a pair of electrodes shown in Figure 6. To test the significance of the dot distribution in the first row, a permutation resampling is applied to each original matrix, and repeated 10,000 times. In each shuffling, the upper-triangle elements are randomly allocated, and a significance test is applied to achieve a *p*-value quantifying the structural difference between the permuted and original matrices. The first three permuted coherence matrices are shown. The averaged *p*-value over the 10,000 permutation tests for the original observations (first row) are all smaller than 10^−5^, revealing a significant difference between the original distribution and its permutations.

To demonstrate that the clustering patterns shown in Figure 6 represent meaningful and consistent changes in network connectivity—and are not simply the outcome of random happenstance—we apply a permutation test to each coherence-change matrix. In this test, we shuffled the elements of the coherence matrix. The null hypothesis is that the permuted coherence matrix could result in an electrode-pair distribution similar to that seen in Figure 6; the alternative hypothesis is that the electrode-pair distribution generated from the permuted coherence matrix is significantly different with the originally observed pattern. A chi-squared statistic is applied in estimating the *p*-value. We first divided the brain into five areas: frontal, occipital, left-temporal, right-temporal, and parietal. The chi-squared distribution index is given by

(4) $$\chi^{2}=\sum_{i=1}^{5}\frac{{\left(E_{\text{original}}^{i}-E_{\text{perm}}^{i}\right)}^{2}}{E_{\text{original}}^{i}}$$

where *E*^*i*^_original_ is the original number of electrodes (i.e., the dot coordinates in the first row of Figure 7) in section *i*; *E*^*i*^_perm_ is the number of permuted electrodes (e.g., the dot coordinates in the second row of Figure 7) in the same section. The *p*-value is calculated by the Matlab command p = 1 - chi2cdf(χ^2^, dof), in which dof (degree of freedom) is set to 4 (dof = number of data category − 1).

After 10,000 permutation tests, all permuted electrode-pair distributions are found to be significantly (*p* < 10^−5^) different from the original one. This statistical result supports our alternative hypothesis that the derived electrode-pair distribution pattern is meaningful and cannot be randomly generated. Actually, visual examination of the first row in Figure 7 clearly reveals genuine dot clusters, the structure of which disappears in the permuted matrices, so it is not surprising that the original data complexity cannot be reproduced from the randomized data distribution. We applied the same statistical test to the coherence matrices corresponding to the patterns shown in Figure 4 and obtained the same result, namely, that the original distribution is significantly different from its permutation resampling.

### 4.3. Comparison with theory: interacting Turing–Hopf induced chaotic slow-waves

A recent theoretical prediction by Steyn-Ross et al. (2013) introduces an interacting Turing–Hopf mechanism as a source for sub-delta slow-waves that emerge during propofol anesthesia. We now give a brief overview of the cortical model; for full mathematical details refer to Steyn-Ross et al. (2013).

The cortex is represented as a set of eight coupled partial-differential equations that describe the mean-field (spatially-averaged) firing activity of populations of excitatory and inhibitory neurons that are uniformly distributed across a two-dimensional sheet of gray-matter cortical tissue. The neural populations communicate locally and at longer ranges via chemical synapses, and also through electrical synapses (gap junctions) that allow direct diffusive currents to flow between adjoining neurons. Inhibitory-to-inhibitory (*i*-*i*) gap-junction connections are abundant and ubiquitous throughout the central nervous system (Bennett and Zukin, 2004). Fukuda et al. (2006) characterized the dendritic gap-junction connections in cat visual cortex as forming “dense and far-ranging networks.” Using the Fukuda measurements, we estimated an upper bound for the per-neuron region of gap-junction influence as an area *D*_2_ ≲ 0.6 cm^2^ (Steyn-Ross et al., 2007), with symbol *D* chosen to indicate a *diffusive* coupling strength. Using a dendritic relaxation time of τ ≈ 40 ms as our time-scale, the ratio *D*_2_/τ defines a diffusion coefficient (with dimensions area/time) for voltage change in the inhibitory population. In contrast to the relative abundance of *i*-*i* gap junctions, evidence for excitatory-to-excitatory (*e*-*e*) diffusive coupling is very sparse (Bennett and Zukin, 2004), so we have set the excitatory coupling strength at an arbitrarily small fraction of the inhibitory value: *D*_1_ = *D*_2_/100. We note that inhibitory diffusive dominance is a prerequisite for the spontaneous formation of Turing structures (Turing, 1952) of spatially-patterned cortical activity.

For the model results reported here, we used the same parameter settings as listed in Table I of Steyn-Ross et al. (2013), apart from the white-matter long-range connections which have been ignored for simplicity.

**Figure 8:**
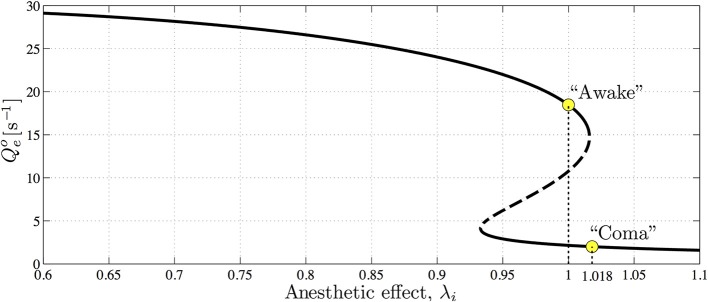
**The steady-state firing rates *Q*^*o*^_*e*_ as a function of varying anesthetic inhibition λ_*i*_ at a particular cortical excitation**. The upper, high-firing and lower, low-firing branches (solid curve) are considered to be “awake” and “coma” states, respectively, with the “coma” state being associated with anesthetic-induced unconsciousness. Dashed curve indicates an unstable branch from which the cortex has the potential to jump to either the upper or lower stable branches. Upper and lower marked circles indicate references at λ_*i*_ = 1.0 and 1.018 on awake and coma branches, respectively. (Figure reproduced from Steyn-Ross et al., 2013).

Figure 8 shows that the steady-state excitatory neuronal firing rates *Q*^*o*^_*e*_ of the model forms a reversed S-shape distribution with the upper branch corresponding to an activated cortical state identified as awake (or REM sleep), and the lower branch corresponding to a suppressed cortical state identified as propofol anesthetic induced coma (or SWS) (Steyn-Ross et al., 2005, 2012). By increasing the concentration of propofol anesthesia λ_*i*_, the model describes the anesthesia-induced transition from consciousness to unconsciousness.

**Figure 9:**
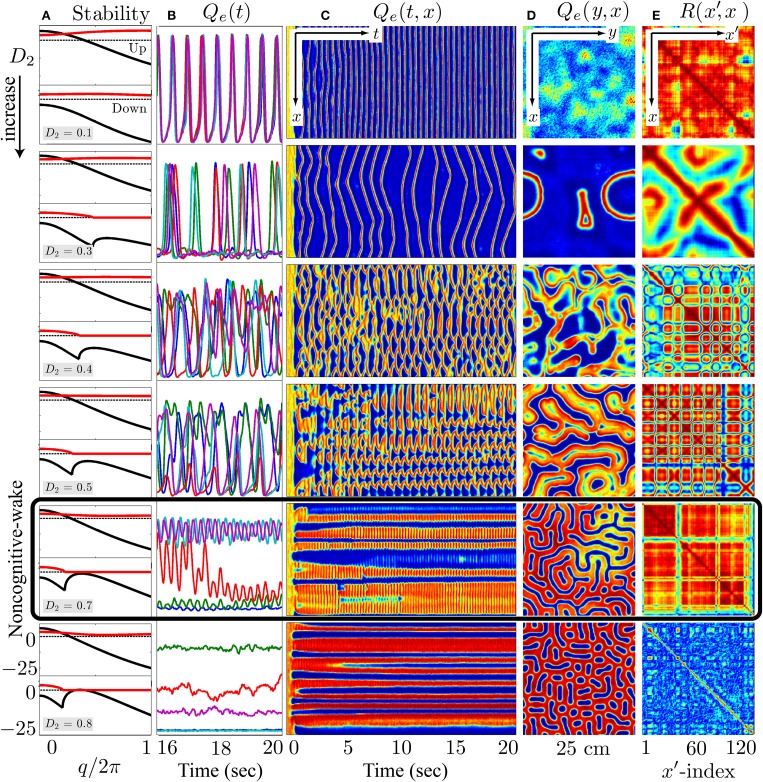
**At the λ_*i*_ = 1.0 wake state, cortical stability analysis and spatiotemporal dynamics for varying gap-junction strength *D*_2_ from 0.1 (top row) to 0.8 cm^2^ (bottom)**. Model cortex is initialized from the top high-firing branch of steady-state manifold marked as “Awake” in Figure 8. **(A)** Cortical stability analysis showing dominant eigenvalue dispersion curve of the real (black) and imaginary (red) parts as a function of scaled wavenumber for top- and bottom-branch equilibria at fixed anesthetic effect λ_*i*_ = 1.0 in Figure 8. Thus, each panel has two parts in it—the upper part corresponds to the top-branch, the lower part to the bottom-branch. The dotted line marks zero. **(B)** Last 4-s time-series of excitatory firing-rate *Q*_*e*_(*t*) extracted from 5 equally-spaced grid-points in **(C)** *Q*_*e*_(*t*, *x*) space-time chart representing the full 20-s time-evolution of cortical activity along the *y* = 60 midline strip; *y*-axis ranges from 0 to 30 s^−1^. **(D)** Bird's-eye snapshot *Q*_*e*_(*y*, *x*) of the cortex when *t* = 20 s. **(E)** Phase coherence map *R*(*x*′, *x*) showing synchronization level of firing-rate between *Q*_*e*_(*t*, *x*) and *Q*_*e*_(*t*, *x*′) for the final 5-s time evolution. The coherence level is computed via Hilbert transform Equation (3) with a transition from *red* to *blue* meaning high to low coherence. In **(C–E)**, color scale from blue to red indicates the numerical range from low to high. (Figure modified from Steyn-Ross et al., 2013).

**Figure 10:**
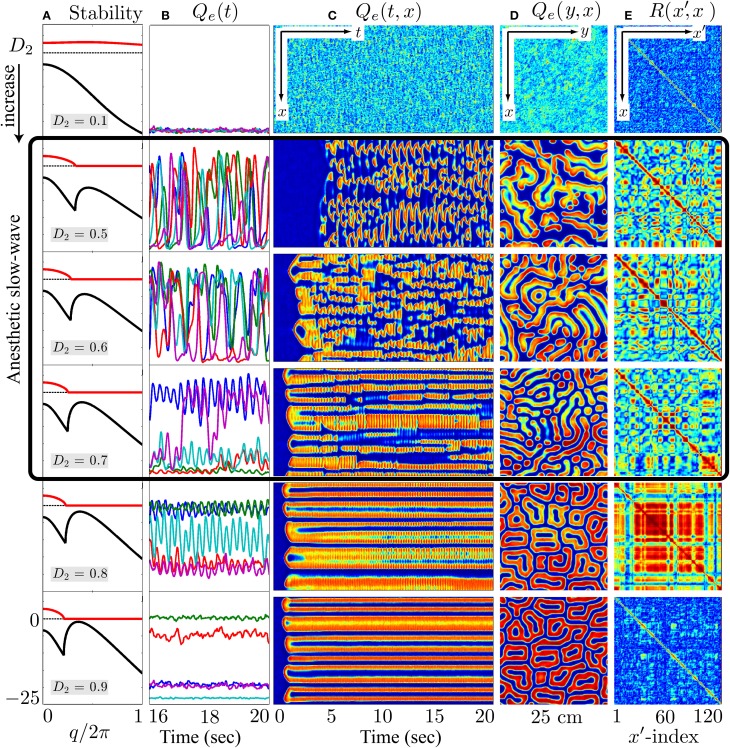
**At the λ_*i*_ = 1.018 coma state, cortical stability analysis and spatiotemporal dynamics of varying gap-junction strength *D*_2_ from 0.1 (top row) to 0.9 cm^2^ (bottom)**. Model cortex is initialized from the bottom low-firing branch of steady-state manifold marked as “Coma” in Figure 8. **(A)** Cortical stability analysis showing dominant eigenvalue dispersion curve of the real (*black*) and imaginary (*red*) parts as a function of scaled wavenumber at anesthetic effect λ_*i*_ = 1.018 in Figure 8. **(B)** Last 4-s time-series of excitatory firing-rate *Q*_*e*_(*t*) extracted from 5 equal-spaced grid-points in **(C)** *Q*_*e*_(*t*, *x*) space-time chart representing the full 20-s time-evolution of cortical activity along the *y* = 60 midline strip. **(D)** Bird's-eye snapshot *Q*_*e*_(*y*, *x*) of the cortex when *t* = 20 s. **(E)** Phase coherence map *R*(*x*′, *x*) showing synchronization level of firing-rate between *Q*_*e*_(*t*, *x*) and *Q*_*e*_(*t*, *x*′) for the final 5-s time evolution. In **(C–E)**, color scale from blue to red indicates the numerical range from low to high. (Figure modified from Steyn-Ross et al., 2013).

Inhibitory gap-junction strength *D*_2_ is treated as a bifurcation parameter controlling the stability and the emergent behavior of the cortical model. The effect of interneuronal gap junctions is to produce diffusion terms similar in form to those found in standard reaction-diffusion models that support Turing structures (Turing, 1952). The cortical dynamics at selected “awake” and “coma” coordinates in Figure 8 with respect to the variation of *D*_2_ were examined by the stability analysis, and numerical simulations are shown in Figures 9, 10.

In the awake cortical simulations of Figure 9, when the gap-junction strength is sufficiently large (*D*_2_ = 0.7 cm^2^), linear stability analysis of the up-branch steady-state at λ_*i*_ = 1 in Figure 8 predicts whole-of-cortex Hopf oscillations; while the down-branch steady-state shows a damped-Hopf at wavenumber *q* = 0 plus a damped-Turing at *q* ≠ 0. The time-series and strip-chart depict a stable Turing–Hopf mode evolution where the cortical Turing patterns oscillate in small amplitudes. Such Turing-interacted Hopf slow-oscillation have been interpreted as representing the resting state of the cortex (Steyn-Ross et al., 2012) or non-cognitive idling state (Steyn-Ross et al., 2011). These slow patterned oscillations may relate to very slow (≤0.1 Hz) fluctuations in BOLD (blood-oxygen-level dependent) signals detected using fMRI (functional magnetic resonance imaging) of relaxed, non-tasked human brains (Fox et al., 2005; Fransson, 2005).

On the other hand, for the anesthetized cortex, anesthetic effect λ_*i*_ = 1.018 is just beyond the multiple steady-states region where the awake cortex stays at the up-branch of λ_*i*_ = 1.0. This subtle change in coordinates means that the cortical stability is guided only by the steady-state at the low-firing bottom branch. In Figure 10, at the closure of the gap-junction *D*_2_ = 0.1 cm^2^, linear stability analysis [column (a)] predicts a heavily damped Hopf, which is consistent with computer simulations of the cortical equations. Most general anesthetics will enhance the strength of the inhibitory postsynaptic potential (IPSP) (Franks and Lieb, 1994; Kitamura et al., 2002), as well inhibit gap-junction communication (Wentlandt et al., 2006). Consequently further increases in *D*_2_ (for *D*_2_ < 0.7 cm^2^ of Figure 10) lead the cortex into a chaotic phase, arising from the competitive interference between Hopf and Turing instabilities. Such mixed instabilities may provide a mechanism for the emergence of turbulent slow-waves of inductive anesthesia, characterized by low phase-coherence. *D*_2_ = 0.7 cm^2^ is the border of the anesthetic slow oscillations; larger values of *D*_2_ (e.g., *D*_2_ = 0.8 cm^2^) rebalances the Turing and Hopf instabilities in favor of spatially structured Turing pattern oscillating at a low Hopf frequency (~3 Hz). Such mixed-mode interference is very similar to the non cognitive-wake cortex at *D*_2_ = 0.7 cm^2^ in Figure 9. Nevertheless, because the cortex is still under anesthetic coma, Steyn-Ross et al. label this coherent oscillation as “anesthetic delirium,” a clinical state common during emergence from general anesthesia and associated with excitability and confusion (Olympio, 1991).

**Figure 11:**
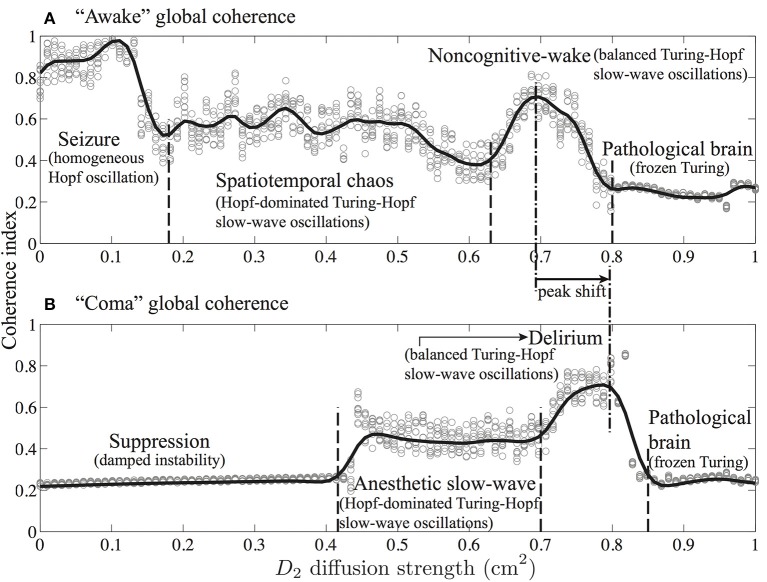
**Global phase-coherence trends with respect to inhibitory strength for the cortex at **(A)** awake (λ_*i*_ = 1) and **(B)** comatose (λ_*i*_ = 1.018) states**. Inhibitory strength *D*_2_ is evenly spaced (0.01 cm^2^ interval) in the range 0.0–1.0 cm^2^. At a given *D*_2_, simulations were repeated 10 times. For each simulation, we first computed the phase-coherence matrix *R*(*x*′, *x*) for the final 5-s time evolution (see Figures 9E, 10E), then extracted its upper-triangular matrix mean as an estimate of global phase-coherence, which is represented as a gray cycle in the figure. The trend curves were produced by spline function in Matlab curve-fitting toolbox. (Figure modified from Steyn-Ross et al., 2013).

Figures 9, 10 indicate that Turing–Hopf interaction dynamics arise from variations in *D*_2_ inhibitory strength. To further track these Turing–Hopf dynamics, Steyn-Ross et al. computed the global coherence of a given *D*_2_ by taking the mean of the upper-triangle of the coherence matrix *R*(*x*′, *x*) defined in Figures 9E, 10E. A comprehensive inspection of the global coherence relating to the inhibitory strength is presented in Figure 11. We see a high global coherence in the non-cognitive state, where the inhibitory diffusion is moderately strong *D*_2_ ≃ 0.7 cm^2^. For the anesthetized cortex, the anesthetic drug shifts the activated “Noncognitive-wake” coherence peak to the right, implying a possible hysteresis effect such that an anesthetized cortex requires a stronger Turing instability to reinforce an activated state. To the left of the peak for the delirium state, there is a broad intermediate zone of *D*_2_ experiencing reduced coherence, which results from large, low frequency chaotic oscillations.

These model results drawn from Steyn-Ross et al. (2013) allow a prediction that the passage from wake to anesthetic unconsciousness should manifest as a decrease in phase coherence between separated cortical electrodes.

## 5. Discussion

Phase-coherence is a measure that quantifies the degree to which the same frequency components of two EEG channels preserve their relative phase over a certain time period. The phase stability between two EEG channels indicates their phase synchronization, reflecting the functional correlations of spatially divergent cortical regions.

In this study, we investigated systematic phase-synchronization changes between pairs of EEG channels in the sub-delta band, during propofol anesthetic induction. An EEG phase-coherence processing algorithm, EEG_coherence, was developed in Matlab and applied to archival EEG data from a group of subjects. EEG_coherence uses the Hilbert transform to extract instantaneous phase-angles from non-stationary EEG signals, and yields a phase-coupling index appraising the phase-shift consistency between pairs of EEG channels. The trends of such EEG coherence change between two brain states are statistically tested via a Mann-Whitney *U*-test, which is a simple non-parametric test without the requirement of a specific data distribution.

Our sub-delta band (≲1.5 Hz) EEG study discloses a regional *decrease* in phase coherence under propofol anesthesia in both the frontal and the occipital cortical areas, and also for electrode pairs that link these two areas. Simultaneously, more strongly phase-coupled neuronal activity is found in the temporal–frontal, temporal–occipital and left–right temporal regions. Such contrasts in coherence change suggest an underlying compensatory mechanism of sub-delta band activity between a subsystem of fronto–occipital and temporal cortical regions. Our findings of reduced-coherence between particular electrode-pairs is similar to clinical reports (Morikawa et al., 1997; Koskinen et al., 2001) where the frontal cortical region exhibits a negative inter-correlation during anesthetic coma.

Such changes in large-scale neuronal coupling may be an anesthetic indicator of unconsciousness when the subject is disconnected from the environment with reduced cognition level. A leading hypothesis suggests that anesthetics cause unconsciousness by disrupting functional connectivity between cortical areas (Mashour, 2004; Alkire et al., 2008). A recent work by Lewis et al. (2012) found that the slow oscillation is a fundamental component of propofol-induced unconsciousness and it occurs asynchronously across cortex, interrupting the cortical integration of information processing. Thus, spatiotemporal slow oscillation dynamics may mediate the fragmentation of cortical networks at both the local and global scale, leading to reduced coherence in neuronal communications. Meanwhile, the presented reduced phase-coherence along the fronto-occipital axis is consistent with an animal study by Imas et al. (2006) that the anterio–posterior coherence in both 5–25 and 26–50 Hz bands was significantly reduced by isoflurane in the rat.

In contrast, Dumermuth and Lehmann (1981) reported a high interhemispheric coherence between the left and right parietal areas with deepening slow wave sleep. They postulated that the high coherence may reflect the interhemispheric transfer of information. Later, research by Mölle et al. (2004) reinforced Dumermuth and Lehmann's findings and verified their hypothesis by comparing coherence changes for subjects during the slow-wave sleep with or without pre-learning tasks. Mölle et al. observed significantly increased coherence during the occurrence of slow oscillations (<1 Hz) for subjects after learning tasks; Figures 1, 2 in Mölle et al. (2004) show increased sub-delta band EEG coherence between the left- and right-temporal regions. This left–right strengthening is concordant with our propofol results shown in Figure 4 (lower panel).

The apparently compensatory weakening of frontal and occipital coherence (upper panel of Figure 4) supports the hypothesis of Steyn-Ross et al. (2013) that propofol anesthesia should induce a decrease in EEG coherence. When there is little or no anesthetic effect, a sufficiently strong inhibitory diffusion (i.e., gap-junction strength) allows a rough balance between Turing pattern and Hopf oscillation instabilities, leading to a slow Hopf oscillations of high global coherence with sustained spatial structure (see *D*_2_ = 0.7 cm^2^ simulation in Figure 9). Such interacting low-frequency Hopf and Turing instabilities may form the substrate for the cognitive state, namely, the “default” background state for the non-cognitive brain during wake. Its slow beating dynamics (≤0.1 Hz) is similar to what is observed in BOLD functional MRI recording of relaxed, non-tasked human brains (Fox et al., 2005; Fransson, 2005).

An increase in anesthetic effect λ_*i*_ suppresses cortical activity, leading to an anesthetized coma state. Here, intermediate values of *D*_2_ are expected since propofol anesthetic will tend to block gap-junctions (Wentlandt et al., 2006) and thus weaken inhibitory diffusion. This will damp the Turing instability, allowing the Hopf instability to become dominant, leading to spontaneous emergence of large-amplitude slow chaotic oscillations (see the highlighted simulations in Figure 10). We note that this dynamical mechanism for the slow oscillation is quite distinct from the conventional view of cyclic alternations in extracellular ionic (Ca^2+^) concentration (Massimini and Amzica, 2001) that may be initiated by tiny clusters of pacemaker neurons in layer-5 of cerebral cortex (Stroh et al., 2013).

The emergent slow oscillation is predicted to be chaotic in space and time, and this is the reason for the expected decrease in phase coherence with descent into anesthetic hypnosis. Therefore, the *increase* in coherence seen in the left–right electrode pairs cannot be explained by the model. A possible resolution for this discrepancy may lie in the model's neglect of a major component of cortical white-matter architecture, namely the corpus callosum that connects left and right hemispheres of the cortex. It is possible that as local independent activity is suppressed during deep anesthesia, the anatomical left–right connectivity becomes *functionally* stronger, thus invalidating the model assumption of a homogeneous cortex. In future modeling work it would be useful to investigate if an imposed left–right cortical connection symmetry might tend to enhance inter-hemispheric coherence while leaving frontal–occipital dynamics unchanged.

## Author contributions

All authors contributed extensively to the work presented in this paper: Moira L. Steyn-Ross, D. A. Steyn-Ross, Marcus T. Wilson and Jamie W. Sleigh developed the cortical model and proposed the stability analysis. Moira L. Steyn-Ross, D. A. Steyn-Ross and Jamie W. Sleigh developed the Hilbert transform-based phase-coherence index. Jamie W. Sleigh provided the EEG data and clinical references relating to the paper. Kaier Wang developed EEG_coherence algorithm and performed EEG processing and analysis. All authors contributed to the writing of the paper.

### Conflict of interest statement

The authors declare that the research was conducted in the absence of any commercial or financial relationships that could be construed as a potential conflict of interest.

## Footnotes

^1^The electrodes map is available at http://psychophysiology.cpmc.columbia.edu/software/CSDtoolbox/tutorial.html

^2^Automatic Artifact Removal toolbox, an EEGLAB plug-in available at http://www.germangh.com/eeglab_plugin_aar/index.html AAR is based on blind source separation (BSS), and, in contrast to methods already available in the literatures (Jung et al., 2000; Faul et al., 2005), is completely automatic since the user is not required to select any critical analysis parameter. AAR uses a second-order-blind-identification (SOBI) algorithm (Belouchrani et al., 1997) to estimate the mixing matrix that separates the EEG sources and artifacts. The advantages of SOBI over other BSS algorithms are detailed in Gomez-Herrero et al. (2006).

^3^An open source EEG processing Matlab toolbox available at http://sccn.ucsd.edu/eeglab/

^4^The lower triangle of the coherence matrix *R*_(*k*,*n*,*m*)_ and the diagonal unit coherence.
